# The role of phospho-ubiquitin in mitochondrial health and diseases

**DOI:** 10.1016/j.jbc.2026.113128

**Published:** 2026-05-07

**Authors:** Kumar Suresh, Christopher Rainville, David E. Sterner, Matthew S. Goldberg, Tauseef R. Butt

**Affiliations:** 1 Department of Research and Development, Progenra Inc., Malvern, Pennsylvania, USA; 2 Department of Neurology, The University of Alabama at Birmingham, Birmingham, Alabama, USA; 3 Killion Center for Neurodegeneration and Experimental Therapeutics, The University of Alabama at Birmingham, Birmingham, Alabama, USA; 4 Department of Neurobiology, The University of Alabama at Birmingham, Birmingham, Alabama, USA

**Keywords:** aging, autophagy, biomarker, mitochondria, mitophagy, neurodegeneration, parkin, PINK1, phospho-ubiquitin, proteasome

## Abstract

Mitochondria play a major role in cellular health, yet their contribution to chronic diseases has been underestimated. Mitochondria are essential for all tissues and are the major source of ATP in high-energy-demand organs such as brain and heart, which consequently are vulnerable to mitochondrial dysfunction. Failure to repair or remove damaged mitochondria contributes to aging and chronic diseases. Cells have evolved quality control mechanisms, including mitophagy to eliminate damaged mitochondria and mitobiogenesis to replenish them. The ubiquitin-proteasome system (UPS) is responsible for removing misfolded proteins, a process that is highly ATP dependent and therefore reliant on mitochondrial function. In turn, damaged mitochondria are eliminated through coordinated actions of the UPS and lysosomal degradation through mitophagy. Many neurodegenerative diseases are characterized by the presence of disease-specific protein aggregates, such as α-synuclein aggregates in Parkinson’s disease and tau neurofibrillary tangles in Alzheimer’s disease. These aggregates impair mitochondrial function, while dysfunctional mitochondria generate reactive oxygen species that further exacerbate proteotoxic stress, creating a pathogenic cycle. This highlights the functional interplay between mitochondria and the UPS. Recent studies have uncovered phosphorylation of ubiquitin at serine 65 by the mitochondrial kinase PINK1 as a key signal of mitochondrial dysfunction. Phospho-Ser65-ubiquitin (pUb) has emerged as an indicator of mitochondrial health and a potential biomarker for aging and neurodegenerative disease. However, due largely to a lack of tools, little is known about the role of pUb in cellular physiology. Here, we review the current landscape of pUb biology, the phospho-ubiquitome, and its role as biomarker for mitochondrial health and neurodegeneration.

## Ubiquitin: more than just a proteasomal signal

Seminal discoveries of the ubiquitin proteasome system (UPS) identified its fundamental role in the selective degradation of short-lived proteins. However, the role of UPS in important aspects of cell physiology other than protein degradation was not initially envisioned. Over the last 2 decades, the role of UPS has been established in regulating numerous cellular processes, such as transcriptional regulation, protein translocation, signal transduction, endocytosis, autophagy, and mitophagy (1, 2, 3, 4, 5). Ubiquitin contains seven lysines on its surface, K6, K11, K27, K29, K33, K48, and K63. Proteomics data from cellular proteins have established that poly-ubiquitin chains are extended from all the lysines. K48-linked poly-ubiquitin chains on target proteins are responsible for proteasomal degradation of proteins. K63-linked poly-ubiquitin chains are primarily involved in endocytosis, autophagy, receptor regulation, signaling, and other nondegradative functions (2, 3, 4). K6- and K11-linked poly-ubiquitin chains are also implicated in proteasomal degradation (3, 5, 6). However, the roles of K27, K29, and K33 chains remain poorly characterized. Mono-ubiquitination of histones is a well-established regulator of DNA replication and transcription; however, its dynamic regulation is still being unraveled (1). The discovery of a new ubiquitin modification, phosphorylation at serine 65 (pUb), has added a new dimension to an already enriched cellular pathway (7, 8). The field of pUb is in its infancy, and the purpose of this review is to encapsulate recent developments and to point out the gaps that exist in our current knowledge.

## Phospho-ubiquitin and mitochondrial quality control

### Ubiquitin is phosphorylated by PINK1 to activate the E3 ligase Parkin

**Figure 1:**
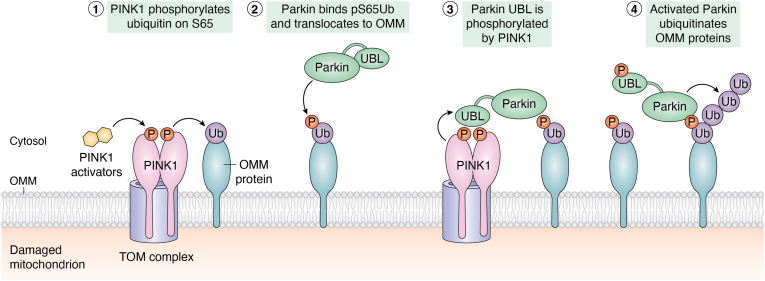
**The molecular basis of PINK1/Parkin activation by phospho-ubiquitin and initiation of mitophagy.** PINK1 is normally degraded and absent from healthy mitochondrial membrane. Under disease state or damage to mitochondria, PINK1 is stabilized on mitochondria and exists as a homodimer associated with TOM complex. Stabilized PINK1 phosphorylates the Ser65 position of ubiquitin attached to an adjacent outer mitochondrial membrane (OMM) protein. Cytosolic Parkin E3 ligase exists in an inactive form as its ubiquitin-like domain (UBL) blocks its activity. Parkin binds to pUb on the OMM; upon binding, the conformational change alters the UBL domain, thus releasing the autoinhibitory state of parkin. PINK1 phosphorylates parkin UBL domain at Ser65, which leads to further structural changes in Parkin. The fully activated Parkin can synthesize poly-ubiquitin chains on OMM proteins, which are further phosphorylated by PINK1. This process is characterized as the feedforward mechanism that jump-starts formation of polyubiquitin and p-polyubiquitin chains on mitochondria. The therapeutic concept of PINK1/Parkin activation is realized by the development of small molecules that activate PINK1-mediated mitophagy. PINK1, PTEN-induced kinase 1; Ser65, serine 65; TOM, translocase of the outer mitochondrial membrane complex; UBL, ubiquitin-like domain.

Ubiquitin is phosphorylated at serine 65 (Ser65) by PTEN-induced kinase 1 (PINK1). Although PINK1 mutations were implicated in early-onset Parkinson’s disease (PD) in 2004, ubiquitin’s role as a *bona fide* PINK1 substrate was only elucidated a decade later (7, 9, 10, 11, 12). Soon after PINK1 was discovered, its role in activating the E3 ubiquitin ligase Parkin was established. Parkin contains a ubiquitin like domain (UBL) at its N terminus (13, 14) which blocks Parkin’s E3 ligase activity *via* intramolecular interactions. Although the Parkin UBL domain exhibits only 30% sequence identity to ubiquitin, its three-dimensional fold is essentially identical (13, 14). Interestingly, PINK1 phosphorylates Ser65 in both the Parkin UBL and ubiquitin (15, 16). Phosphorylation of Parkin UBL unblocks the Really Interesting New Gene domain (RING2) of Parkin that is responsible for ligating ubiquitin to mitochondrial outer mitochondrial membrane (OMM) proteins (17, 18, 19). Remarkably, pUb also binds to the Parkin RING1 domain to further activate Parkin’s E3 ligase activity (17, 20, 21). PINK1/*PARK6* gene KO studies in cell and animal models suggest that PINK1 is the only kinase that phosphorylates ubiquitin at Ser65 (22, 23). Phospho-poly-Ub levels are significantly reduced in Parkin/*PARK2* KO mice and cells (23). These findings demonstrate that PINK1 and Parkin are the key regulators of mitophagy, and pUb is a pivotal player in the scheme (Fig. 1).

### Mitophagy is dependent on a feedforward loop driven by PINK1 and Parkin

Mitophagy is mitochondria-specific autophagy that preserves mitochondrial efficiency including ATP, lipid, and heme synthesis (24, 25). Recognition of impaired mitochondria triggers phosphorylation and ubiquitination of OMM proteins, marking them for degradation and initiating lysosomal clearance of the defective organelles (24, 25). This signaling is driven through coordinated efforts of mitochondrial damage sensors and signaling effectors, predominantly PINK1 and Parkin, respectively. Loss-of-function mutations in either gene cause early-onset autosomal recessive juvenile PD (ARJPD) (26), implying a causal role for mitophagic failure in ARJPD.

In a healthy cell, PINK1 is normally targeted to mitochondria and cleaved by the mitochondrial transmembrane protease PARL, but the kinase domain released into cytosol is rapidly ubiquitinated through the N-end-rule E3 ligases and degraded by the proteasome (27). Upon mitochondrial damage PINK1 is stabilized on the TOM complex of OMM, where PINK1 homodimerizes and each monomer phosphorylates the other monomer in the dimer (28). The activated PINK1 phosphorylates Ser65 of ubiquitin present on OMM proteins (7, 12, 29, 30) leading to the translocation of cytosolic Parkin to the OMM because of Parkin’s ability to bind pUb with high affinity (31). Indeed, several amino acid substitutions in the pUb-binding interface of Parkin (K151, G284, H302, R305, and A320) hypothesized to disrupt the Parkin-pUb interaction impede Parkin activation and recruitment to the mitochondria, and several more substitutions (L283P, H279P, R275W, Q311H, and G328E) have been associated with ARJPD or cancer (19, 21, 32, 33). Recognition of pUb by the Parkin RING1 domain and subsequent direct phosphorylation of Parkin UBL by PINK1 leads to the release of Parkin’s autoinhibited conformation, further enhancement of pUb binding, and complete activation of Parkin (7, 12, 32, 34, 35). Activated Parkin promiscuously ubiquitinates multiple proteins, including MFN1, MFN2, TOM70, VDAC1, BAK, FIS1, and TOM20 (36). The newly-ubiquitinated OMM proteins then serve as additional substrates for ubiquitin phosphorylation by PINK1 (Fig. 1), resulting in a feedforward loop that drives mitophagy (18, 37, 38).

**Figure 2:**
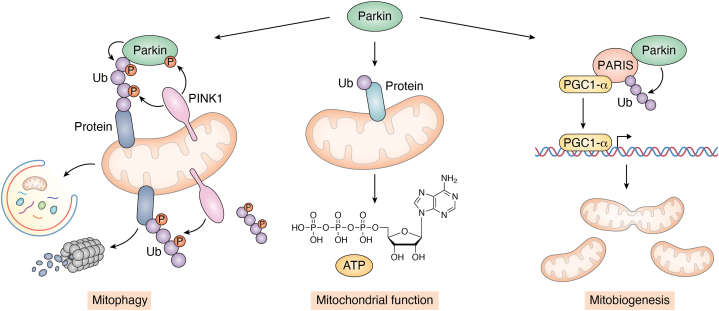
**Ubiquitin, the proteasome, mitophagy, and mitobiogenesis.** Mitochondrial depolarization and dysfunction results in stabilization and activation of PINK1, which then phosphorylates ubiquitin conjugated to OMM proteins at the Ser65 residue. pUb serves as a hotspot for recruitment of Parkin E3 ligase to mitochondria. PINK1 then phosphorylates Parkin at the Ser65 residue in its UBL domain, leading to Parkin activation and subsequent generation of copious amounts of pUb chains on damaged mitochondria. These pUb-containing chains are recognized by autophagy receptors, leading to engulfment and degradation of damaged mitochondria by mitophagy. Activation of the Parkin/PINK1 pathway can also promote mitochondrial function and increase ATP production. In addition, activated Parkin ubiquitinates and causes proteasomal degradation of PARIS, a repressor of PGC1α, the major mitochondrial transcription factor. Derepressed PGC1α promotes mitochondrial gene transcription and mitobiogenesis. OMM, outer mitochondrial membrane; PINK1, PTEN-induced kinase 1; pUb, Ser65-phosphorylated ubiquitin; Ser65, serine 65; UBL, ubiquitin-like domain.

Beyond mitophagy, pUb signaling has also been shown to regulate mitobiogenesis. Activated parkin poly-ubiquitinates PARIS, a repressor for PGC1-α (39, 40) which is a global activator of nuclear mitochondrial gene transcription (Fig. 2). Thus, PINK1/parkin signaling not only initiates the removal of damaged mitochondria but also promotes mitobiogenesis. It is also known that pUb can be incorporated into poly-ubiquitin chains by ubiquitin ligases other than parkin *in vitro* (8). Whether pUb and PINK1 play a role in other aspects of cell physiology remains an open question.

### Phospho-ubiquitin is both a substrate and product of PINK1/parkin-mediated mitophagy

As PINK1’s primary phosphorylation target, pUb is one of the earliest detectable markers of mitochondrial dysfunction and mitophagy (7, 20, 21, 34). pUb regulates the mitophagy process through direct interactions with the autophagy receptors optineurin (OPTN) and NDP52 (38). Many of the OMM substrates ubiquitinated by Parkin in response to mitochondrial damage in mammalian cells are degraded by the proteasome, indicating that polyubiquitin chains containing pUb can be targeted for proteasomal degradation. In fact, p-poly-ubiquitinated protein is degraded by the proteasome with slower kinetics (41). Studies using a phosphomimetic ubiquitin (S65E) mutant in yeast also indicated decreased proteasomal protein degradation (42). These studies suggest potential roles of pUbs beyond mitophagy. Although the precise functional differences between pUb and unphosphorylated Ub are still under investigation, pUb does alter the structure of ubiquitin as well as ubiquitin recognition and activity of some E2, E3, and deubiquitinase (DUB) proteins, which have been reviewed thoroughly (43). The proteasome and lysosome have been shown to cooperatively contribute to pUb turnover, but a full mechanism remains to be characterized (44). Like other protein phosphorylation events, ubiquitin phosphorylation is reversible, and the phosphatases PTEN-L and PPEF2 can dephosphorylate pUb (45, 46). The function of pUb phosphatases in mitophagy remains to be elucidated. The majority of cellular pUb exists as either monoubiquitin or polyubiquitin, and mitophagy adapter proteins are capable of recognizing ubiquitin chains assembled through the K6, K11, K48, and K63 residues (47). In particular, K63-linked chains are elevated in response to mitochondrial damage in neurons. Interestingly, in *Drosophila melanogaster*, oxidant exposure led to the PINK1-dependent selective upregulation of K6 chains, but not K11, K48, K63 chains or total Ub levels (44). These observations indicate the existence of diverse and context-specific chain selective p-poly-ubiquitin recognition motifs in cells.

Phosphorylation of ubiquitin at residues Thr12 and Ser57 has been detected (48, 49). However, their biological functions and relevance to Parkin activation remain largely undefined. PINK1 is the only known kinase of Ser65 on Ub, making pUb a strong proxy for PINK1 activity and mitophagy (50). pUb makes up <0.1% of the total Ub in healthy human cells (up to 2% of the total Ub pool and 20% of mitochondrial Ub in cells with depolarized mitochondria) (43). In the following sections, we will examine the relationship between mitophagy, neurodegenerative disease, and pUb.

### PINK1 and Parkin independent mitophagy

Although the PINK1/Parkin-mediated mitophagy pathway is the most well-studied, multiple PINK1/Parkin-independent mitophagy pathways have also been described. Mice deficient for PINK1 or Parkin have generally mild phenotypes, which are characterized by differences in mitochondrial morphology or quality control (51, 52). This suggests that other proteins have functions that overlap at least partly with PINK1 and Parkin. MUL1 (a mitochondrial-associated E3 ligase) and the E3 ligase ARIH1 can associate with damaged mitochondria and compensate for Parkin to ubiquitinate mitochondrial proteins and induce mitophagy (53). In HeLa cells that do not express Parkin, expression of ARIH1 ligase can induce mitophagy after phosphorylation by PINK1 (54). Receptor-mediated mitophagy pathways rely on OMM proteins with LC-3 interacting regions such as BNIP3, NIX, FUNDC1, BCL2L13, FKBP8, and AMBRA1 (55, 56, 57, 58, 59, 60). BNIP3 and NIX/BNIP3L promote interaction with autophagy receptor LC3B (61) inducing mitophagy when overexpressed in HeLa cells. McWilliams *et al.* have analyzed the extent of mitophagy in mice expressing the mito-QC mitophagy reporter transgene, on both WT and PINK1 KO genetic backgrounds (62). The authors showed that *in vivo*, basal mitophagy is most prominent in highly metabolic cells such as dopaminergic neurons in the substantia nigra, photoreceptor neurons in the retina, and acinar cells in the pancreas. Surprisingly, they found that mitophagy occurs independently of PINK1 in some tissues. In fact, an unexpected elevation of mitophagy was observed in endocrine islet cells in PINK1 KO animals compared with WT, suggesting compensatory activation of receptor-mediated mitophagy (62). These results revealed that PINK1 is not required for basal mitophagy and the extent of mitophagy depends on the level of metabolic activity. In tissues that are highly metabolically active, such as brain, muscle, and pancreas, alternative pathways for mitophagy might take over in PINK1 KO mice. Although alternate mitophagy pathways have been well documented, their roles in neuronal homeostasis and neurodegenerative disease remain poorly characterized. The question arises as to why PINK1/Parkin autosomal recessive mutation populations have such high penetrance of early onset PD if alternative mitophagic pathways are present. Notably, NIX-mediated mitophagy was shown to protect an elderly homozygous Parkin mutation carrier who remained asymptomatic for PD despite the loss of functional Parkin (63). The functional interplay between ubiquitin/p-Ub-mediated and receptor-mediated mitophagy pathways may ensure robust mitochondrial quality control, and defects in this coordination may promote mitochondrial dysfunction in disease.

## Mitochondrial turnover and aging

**Figure 3:**
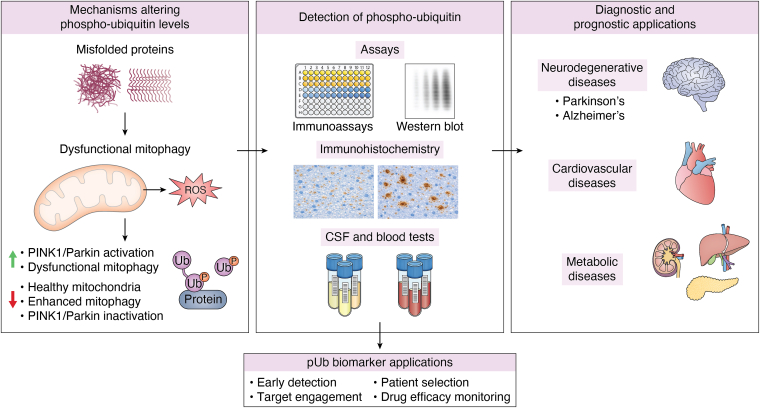
**Phospho-ubiquitin (pUb) as a biomarker: mechanisms, detection modalities, and disease applications.** This figure illustrates key cellular processes that influence pUb abundance, including protein aggregation, mitochondrial dysfunction, and enhanced reactive oxygen species (ROS) generation, altered PINK1 kinase/Parkin activity, mitophagy, and lysosomal blockade. Together, these perturbations (*green arrow* indicates increased pUb and *red arrow* indicates decreased pUb) impact pUb and contribute to disrupted proteostasis and stress-response signaling. Methods for detecting pUb are shown, including immunoassays (*e.g.*, ELISA-based detection), Western blot analysis, immunohistochemistry, and measurement of p-Ub levels in cerebrospinal fluid (CSF) or blood. Diagnostic and prognostic applications of phospho-ubiquitin are highlighted, with relevance to neurodegenerative diseases such as Parkinson's disease and Alzheimer’s disease, as well as cardiovascular disease and metabolic disease, in which impaired mitochondrial quality-control pathways are increasingly recognized contributors to pathophysiology. Collectively, pUb serves as a potential disease indicator, prognostic marker, and therapeutic efficacy indicator across multiple pathological contexts. PINK1, PTEN-induced kinase 1.

There is a wide range of diseases associated with imbalanced mitochondrial regulation, most prominently neurodegenerative diseases (Fig. 3); however, impaired mitochondrial dynamics are well known as a hallmark of typical aging, and possibly even a contributing factor to the aging process itself (64). Age-dependent decline in mitochondrial function has been known for over 60 years, and yet the full relationship between mitochondrial defects and aging remains elusive (65). Impaired mitochondrial turnover has been linked to aging-associated processes, including cellular senescence, generalized inflammation, reduced cellular ATP levels, and decline in stem cell activity (64). Although these processes are known to occur in healthy cells over the course of normal aging, they can be exacerbated through accumulation of misfolded protein aggregates in cells, a typical hallmark of neurodegenerative diseases. In the following sections, we will examine the relationship between pUb, impaired mitophagy, and neurodegenerative diseases.

## Mitophagy in neurodegenerative diseases

### Defective mitophagy is a hallmark of neurodegenerative diseases

Mitophagy is essential for maintaining mitochondrial quality control, particularly in postmitotic neurons that rely heavily on oxidative phosphorylation and energy homeostasis; when mitophagy is impaired, damaged mitochondria accumulate, leading to increased oxidative stress, bioenergetic failure, and neuronal vulnerability. Studies across major neurodegenerative disorders, including PD, Alzheimer’s disease (AD), Huntington’s disease (HD), and ALS consistently document abnormalities in mitophagy pathways and mitochondrial turnover (66, 67, 68). Impairments in PINK1/Parkin-mediated mitophagy are implicated in familial and sporadic PD, and accumulation of defective mitochondria is observed in affected neurons. Likewise, evidence in AD models and human samples links reduced mitophagic capacity with enhanced mitochondrial dysfunction and pathological protein aggregation, while ALS and HD models show disrupted autophagic recognition and clearance of damaged mitochondria contributing to neuronal degeneration. Collectively, these findings support the concept that defective mitophagy and consequent mitochondrial pathology are hallmark mechanisms underlying neurodegenerative disease processes.

### Mitophagic dysfunction in PD

The most prominent neuropathological hallmarks of PD include loss of dopaminergic neurons and the presence of Lewy neurites composed largely of aggregated α-synuclein protein. Lewy body structures also contain mitochondrial fragments, suggesting an interaction of aggregated α-synuclein with mitochondria (69). Consistent with this, iPSCs derived from patients with PD caused by mutations in α-synuclein show delayed mitophagy (70, 71). Treatment of primary neurons with pathogenic α-synuclein preformed fibrils induced defects in mitochondrial respiration, impaired mitophagy, and increased pUb levels (72). Mutations in PARK2 (encoding Parkin) or PARK6 (encoding PINK1) cause ARJPD marked by prominent loss of nigral dopaminergic neurons (26), indicating that the PINK1–pUb–Parkin mitophagy pathway is essential for the survival of these cells. PINK1 being the mitochondrial damage sensor sits at the top of this pathway, implying a crucial role in onset and progression of the neurodegeneration underlying the major clinical symptoms of PD.

Accumulation of pUb occurs adjacent to but not within Lewy bodies, and pUb-positive granules colocalize with mitochondrial markers, suggesting that pUb can be used as a readout of mitochondrial damage and mitophagy alterations in PD patients (73). In support of this possibility, one study identified positive pUb signal in approximately three times as many TH-positive (dopaminergic) neurons as TH-negative neurons (74). PD patients show progression-dependent changes in pUb signal, with high coexistence between pUb and pathological PD hallmarks in early stages, but decreased coexistence with progression (75). This trend culminates in notably absent pUb signal from autopsied brain tissue and primary cells from PD patients bearing *PARK6*/PINK1 mutations (23, 73). pUb levels were elevated in the cerebral cortex of mutator mice, which harbor accelerated accumulation of mitochondrial DNA mutations, compared with WT mice (76). Notably, this increase was not associated with dopaminergic neuron loss and was not further augmented by concomitant Parkin deletion (44). In human subjects with disease-causing missense mutations or multiplications of SNCA (the gene encoding α-synuclein), the level of pUb was significantly increased and correlated with α-synuclein pathology in each brain region analyzed (77). In fact, pUb was shown to track exclusively with LB pathology found in LBD^mut^ carriers. Transgenic mice expressing high levels of human α-synuclein also showed significantly increased pUb in the brain compared to control mice (77).

### Mitophagy dysfunction in AD

Mitophagy is critical for neuronal health, and its impairment has emerged as a key contributor to AD pathogenesis. Postmortem analyses of human hippocampal tissue and studies using iPSC-derived AD neurons demonstrate accumulation of damaged mitochondria, decreased PINK1/Parkin signaling, and reduced autophagic flux, indicating that mitophagy is compromised in AD-affected brains (67, 78, 79, 80, 81, 82). This deficit correlates with early synaptic dysfunction and energy failure, underscoring the vulnerability of postmitotic neurons to mitochondrial stress. The accumulation of amyloid-β (Aβ) and hyperphosphorylated tau contributes directly to mitochondrial dysfunction (83, 84). Aβ oligomers induce reactive oxygen species (ROS) production, promote mitochondrial fragmentation, and impair mitochondrial membrane potential, creating a vicious cycle of oxidative stress and mitophagy inhibition (78, 85, 86, 87, 88). Similarly, hyperphosphorylated tau disrupts mitochondrial transport and recruitment of mitophagy machinery, including PINK1, Parkin, and OPTN, further compromising mitochondrial clearance (83, 89). Several studies have highlighted defects in the PINK1/Parkin pathway as a mechanistic basis for impaired mitophagy in AD (82). In mouse models expressing mutant APP/PS1 or tau, recruitment of Parkin to mitochondria is reduced, leading to accumulation of dysfunctional organelles and increased oxidative stress (78, 90, 91). Enhancing PINK1/Parkin-dependent mitophagy in AD mouse models pharmacologically or genetically not only reduces Aβ and tau burden but also rescues cognitive deficits (78). This provides key proof-of-concept that augmenting PINK1/Parkin-mediated mitophagy can alleviate both neuropathology and neurodegeneration that underlies AD-related cognitive decline.

In addition to mitochondrial defects in AD neurons, enlarged endosomes, accumulation of autophagic vesicles, and reduced lysosomal proteolytic activity are consistently observed in both human AD brains and mouse models of AD, indicating that mitophagy is blocked downstream of autophagosome formation (92, 93, 94). This lysosomal bottleneck could contribute to the accumulation of damaged mitochondria and pathological proteins, amplifying neurodegenerative cascades. Mitochondrial fragmentation, elevated ROS, and the mitophagy marker pUb appear early in AD, preceding overt Aβ plaque deposition and neurofibrillary tangle formation (78, 79, 95). Deficiency in PINK1 expression is associated with cerebral accumulation of Aβ, mitochondrial abnormalities, cognitive impairment, and defective synaptic plasticity, while restoration of PINK1 function reduces Aβ and pTau levels, oxidative stress, and mitochondrial and synaptic dysfunction (78, 96, 97). Dysfunctional mitochondria can trigger improper processing of the Aβ precursor protein APP and pTau, leading to their aggregation and accumulation (79, 98). Strategies aimed at restoring PINK1/Parkin activity, promoting autophagosome-lysosome fusion, or enhancing mitophagic flux have shown promise in preclinical models, reducing pathological protein accumulation and improving cognitive outcomes. Stimulation of PINK1-mediated mitophagy is sufficient to reverse cognitive decline in a *C. elegans* model of AD and hyperphosphorylated tau in human neuronal cells, implying a neuroprotective role for PINK1, likely occurring through pUb-based signaling (78).

### Mitophagic failures in HD

HD is a relatively rare neurodegenerative disorder characterized by the presence of repetitive N-terminal glutamine residues, encoded by a variable number of (37+) CAG trinucleotides in the protein Huntingtin (HTT) (99, 100, 101). HD presents with protein aggregation, motor dysfunction, cognitive processing issues, and psychological symptoms owing to atrophy of GABAergic neurons in the striatum and cerebral cortex, among other regions (99, 100). HTT is a large protein and there is a wide variety of proposed mechanisms for HD onset and progression, including regulation of transcription, as well as defects in the UPS, autophagy, and synaptic transmission (102, 103, 104, 105). Of particular relevance to mitophagy, HTT is known to serve as a scaffold for stabilization of autophagic protein complexes at the cargo recognition stage (106). Furthermore, HTT interacts with the motor proteins, dynein and kinesin, which are critical for transport of endosome/autophagosome/lysosome molecules (107, 108). Most mitochondrial dysfunction in HD, however, appears to stem from the binding and inactivation of the master mitochondrial regulator PGC-1α by mutant HTT (109). Interestingly, mutant HTT can also bind to the valosin-containing protein complex also known as p97, a cellular machinery primarily associated with degrading ubiquitinated ER membrane proteins, and cause its accumulation, thereby inducing excessive mitophagy through a PINK1/Parkin-independent process that results in mitochondrial depletion (110).

### Mitophagic failures in ALS

Most cases of ALS are sporadic, rather than familial, complicating the genetic interrogation of the mechanism of protein aggregation and neurodegeneration in ALS. From the ∼10% of familial ALS cases, however, several mitophagy-related genes have emerged as ALS-linked, including OPTN, p62/SQSTM1, and TBK1 (47, 111). Of the ∼90% of sporadic ALS cases, only ∼10% occur with mutations known to be associated with ALS, leaving ∼80% of ALS cases without a known genetic trigger (112). In the absence of known genetic causes of sporadic ALS, the disease must be examined through functional cellular defects matching known causative mutations, including those in RNA metabolism (*TARDBP* and *FUS*), autophagy/mitophagy (*OPTN*, valosin-containing protein, *SQSTM1*, and *UBQLN2*), and ROS tolerance (*SOD1*) (112). Strikingly, five of the nine genes best-characterized as ALS-causative are involved in mitophagy and ROS tolerance, including the second most-often mutated gene in ALS, *SOD1* (112). Of the remaining four genes, ALS-associated mutant *FUS* is known to reduce mitochondrial size (113), PFN1 is essential for mitochondrial fusion (114), C9ORF72 stabilizes assembly of mitochondrial complex I and regulates autophagosome formation *via* phosphorylation of phagophores (115), and TDP43 downregulates transcription of *PARK2*/Parkin, while triggering an accumulation of nonfunctional cytosolic PINK1 through a reduction in proteasomal activity (116). The unifying factor among ALS-causative mutations is acquired defects in mitochondrial function and turnover, strongly suggesting a neuroprotective role for mitophagy that extends to ALS. Indeed, the ALS-causative mutation *OPTN* E478 G disrupts OPTN-mediated autophagosome recruitment, a key step in mitophagy (117).

## Phospho-ubiquitin as a biomarker

### A need for early and selective biomarkers of neurodegenerative disease

At present, the neurodegeneration field is underserved in biomarkers of preclinical disease. The most definitive biomarkers for PD and AD are aggregated proteins, such as α-synuclein, Aβ, and hyperphosphorylated Tau (118, 119, 120). A new plasma-based biomarker testing platform, Lumipulse, has been launched by Roche and Fujirebio that features a pTau217/βeta-amyloid 1 to 42 plasma ratio test (121, 122). Although these biomarkers are reliable hallmarks of AD, they are difficult to detect in patients until the diseases have progressed to clinical stages (123, 124). Complicating the matter, symptoms can vary from patient to patient, and multiple neurodegenerative diseases can simultaneously affect a single patient (125, 126). Furthermore, up to 30% of clinical neurodegenerative disease diagnoses are inaccurate when compared with autopsy diagnoses, underscoring a need for reliable and reproducible disease screening tools (127, 128, 129). For these reasons, the gold standard of neurodegenerative diagnostics is a postmortem neuropathological autopsy. Whereas biomarkers and PET ligands are available to monitor disease progression in AD, no validated blood-, imaging-, or physiology-based biomarkers exist for PD that can definitively confirm diagnosis or disease stage (130, 131). Binary assays for α-synuclein aggregation are available, but require invasive CSF sampling and cannot differentiate between disease stages or other synucleinopathies with the necessary precision (131, 132). It is therefore critical to identify blood-based biomarkers that are specific to disease state, detectable in the early stages of disease, and tractable for high-throughput analysis in the clinic.

### Connecting the dots: pUb as a biomarker in diagnostics and therapeutics

Various mitochondrial insults activate PINK1, supporting its role as a sensor of mitochondrial damage (133). Because pUb is generated primarily during PINK1/Parkin-mediated mitophagy, basal PINK1 and pUb levels are extremely low, making pUb a potentially selective readout of mitophagy activity (22, 23, 134, 135). Mitophagy research often involves chemical induction of mitochondrial stress, which in turn activates PINK1/Parkin function. The absence of reliable tools to monitor pUb-marked or p-poly-ubiquitinated proteins in normal physiological conditions has hampered progress in studying the role of PINK1 and Parkin in a nonstressed environment. Aggregation of misfolded proteins depolarizes mitochondria, in turn activating PINK1/Parkin signaling. Pathogenic protein aggregates such as α-synuclein (PD) (71), tau protein (AD) (136), HTT (HD) (137), and TDP43 (ALS) (138) are known to impair mitochondria. It is also evident that mitochondrial dysfunction, such as mutations in respiratory chain genes, lead to pathological state (29, 61, 62, 63, 64, 65).

Parkin/PINK1 signaling plays a major role in preventing loss of dopaminergic neurons in inherited forms of PD (130). Dopaminergic neurons are particularly susceptible to mitochondrial dysfunction and damage, and mutations in the genes encoding PINK1 and Parkin manifest as early-onset PD. Remarkably, pUb signal has been shown to correlate with hallmarks of PD, such as Lewy body formation (79). Pathological deposition of α-synuclein triggers mitochondrial dysfunction and mitophagy failure, leading to accumulation of pUb both in primary neurons and *in vivo* (72). In addition, pUb levels can increase due to direct mitochondrial damage by environmental toxins, alterations in autophagosomal/lysosomal/proteasomal function, and changes in PINK1 or Parkin activity. Recent work shows that pUb can be detected in human plasma using an MSD ELISA, and levels are reduced in carriers of the PINK1 Q456X mutation (139), highlighting the potential of pUb as a clinically tractable PD biomarker (53). It is interesting to note that the level of pUb is high among the PD population and low among control populations (74, 130, 140). However, studies using large cohorts are necessary to determine if pUb level can adequately discriminate between PD and control populations.

Interestingly, elevated pUb levels were identified in autopsied brains from Lewy body disease cases but not multiple system atrophy (MSA) cases, suggesting that pUb may be useful as a diagnostic biomarker even to distinguish between highly similar synucleinopathies (77). The difference in pUb levels between Lewy body disease and MSA could be due to differences in the cell type containing the pathological aggregates (neuronal *versus* glial cells) as well as differences in level of PINK1/Parkin expression or activity. At least in the case of Lewy body carriers, the multiplication of SNCA was correlated with elevation of Parkin levels (77). A regulatory dynamic could be envisioned wherein pUb levels are low in healthy individuals, but rise in early to mid-stage disease, positioning pUb level as a potential biomarker for PD onset and progression (23, 73, 75). Aggregated forms of α-synuclein, Aβ, and tau have emerged as biomarkers for PD and AD, respectively. Similarly, imaging studies such as PET scans have been used to detect aggregated forms of proteinopathies (141). Integrating pUb with well-characterized tissue- or disease-specific markers may enhance its diagnostic and prognostic value as a biomarker.

pUb is also promising as a potential biomarker for AD. In autopsied AD patients, pUb signal is notably elevated in the frontal cortex (139). In another study, pUb levels were increased in AD-affected brains, strongly correlating with granulovacuolar degeneration and phosphorylated Tau deposits (79). Interestingly, pUb levels did not correlate with Aβ plaques or mature tau tangles, suggesting that pUb may be useful as an early-stage biomarker for AD (79). Intriguingly, the mRNA abundance of mitophagy-relevant genes is significantly altered in the blood of AD subjects *versus* healthy controls, lending credence to the potential utility of pUb as a blood-based biomarker for AD (142). The rates of sporadic AD, PD, and ALS incidence (and to a lesser extent, HD) increase with age, possibly owing to reduced capacity for overcoming mitochondrial insult (122, 123, 124, 125). pUb levels increase in primary neurons under mitochondrial stress in a PINK1-dependent manner, and elevated pUb was also observed in postmortem brain tissue from PD patients, underscoring the relationships between PINK1-mediated mitophagy, aging, and neurodegeneration (74).

Although UPS can quickly remove misfolded proteins, removal of aggregates requires lysosomal degradation (4, 143, 144). Given that α-synuclein and tau aggregates are ubiquitinated and localize to mitochondria, it is tempting to speculate that they may also carry phospho-polyubiquitin modifications. It is not clear whether sporadic or late-stage PD arises from a failure of ubiquitination machinery and/or lysosomal dysfunction that impairs clearance of pathogenic aggregates and damaged phospho-poly-ubiquitinated mitochondria. Data from human PINK1/Parkin mutant population (familial) indicates that the dysfunction is likely due to the inability of UPS and PINK1/Parkin to initiate the mitophagy process. Answers to these questions will emerge as drugs that activate PINK1/Parkin signaling are tested in human clinical trials. Interestingly, treatment with the putative PINK1 activator MTK458 in mice lead to a reduction in pUb levels in the brain and plasma (72). This reduction in pUb level was attributed to the clearance of damaged mitochondria *via* mitophagy. It is likely that pUb levels are inversely proportional to completion of the mitophagy process. Therefore pUb can be used as a pharmacodynamic marker for impaired mitophagy in neurological disorders (72) (Fig. 3). Whereas small molecule PINK1 activator has entered clinical trial, activators for Parkin remain at preclinical stage (145, 146, 147). These molecules may be combined with therapeutics that promote mitophagy downstream of the PINK1/Parkin pathway and improve lysosomal functions. Because mitophagy is implicated in numerous diseases, mitophagy-activating drugs may have therapeutic potential well beyond PD.

Indeed, poly-ubiquitin signaling is intricately related with autophagy and mitophagy, two processes which are often disrupted in neurodegeneration and protein aggregation-based diseases (91, 98, 99, 108, 148, 149, 150, 151, 152). However, defective mitophagy has been implicated in numerous nonneurological diseases, including metabolic diseases, diabetic retinopathy, myocardial infarction, cardiac hypertrophy, muscular dystrophy, liver disease, autism, epilepsy, schizophrenia, bipolar disorder, depression, obesity, and inflammation, among many other diseases (61, 153) (Fig. 3). Therefore, to benefit from the role of pUb in human blood as a biomarker, pUb may need to be combined with other diagnostic markers.

### Unique poly-ubiquitin chain structures and complexity of the phospho-ubiquitin code

**Figure 4:**
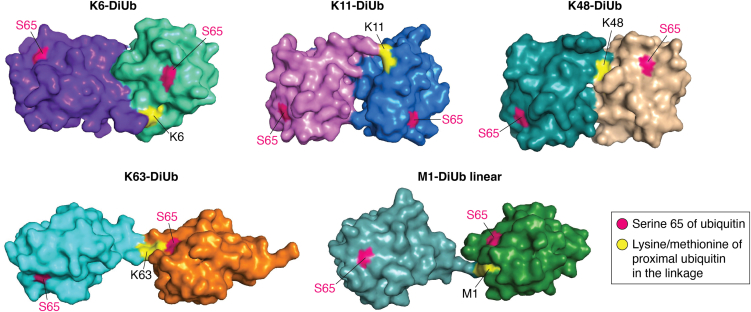
**Diubiquitin structures adopt varying conformations.** Structures of K6 (pdb: 2XK5), K11 (pdb: 2XEW), K48 (pdb: 3AUL), K63 (pdb: 3H7P), and M1-linked (pdb: 2W9N) diubiquitins are depicted, with the position of Ser65 on both distal and proximal ubiquitins labeled in *pink* color. The lysines of proximal ubiquitin involved in the linkages are shown in *yellow* color. The figures are generated using Pymol software v2.5.4.

Although the ubiquitin protein is highly conserved among eukaryotes, various poly-ubiquitin structures provide an insight into the complexity of the ubiquitin code. Remarkably, yeast lacks the PINK1 kinase and therefore does not possess the canonical phospho-Ser65 ubiquitin signaling (154). Poly-ubiquitin chains structures and phospho-Ser65 ubiquitin structures are remarkably conserved in higher eukaryotes. It is important to briefly examine the diversity of poly-ubiquitin chains structures and the impact of pUb. The C-terminus of ubiquitin (Leu-Arg-Gly-Gly) is quite flexible, allowing the ubiquitin polymer to take many shapes (155). Examination of the proposed K6-, K11-, K48-, and K63-linked diubiquitin structures provide a glimpse into the molecular basis of interaction between the two adjacent ubiquitin monomers (Fig. 4). The K63-linked diubiquitin has an open structure at the isopeptide bond, while the K48-linked diubiquitin is a compact structure (Fig. 4). These structures are distinct from the K6- and K11-linked diubiquitin (Fig. 4). Phosphorylation at Ser65 can alter the surface of ubiquitin as well as introduce a conformational change leading to a minor conformation with the β5-strand and C-terminal tail shifted relative to the β-sheet core of the ubiquitin structure (8). Studies suggest that there is no difference between native ubiquitin and pUb charging by E1 activating enzyme and E2 conjugating enzymes (8). It appears that p-poly-ubiquitin chains are somewhat resistant to cleavage by multiple DUBs: at least 10 out of 12 DUBs are impaired in cleaving p-polyUb chains (8). Thus, pUb containing chains on mitochondrial proteins ensure efficient removal of damaged mitochondria.

**Figure 5:**
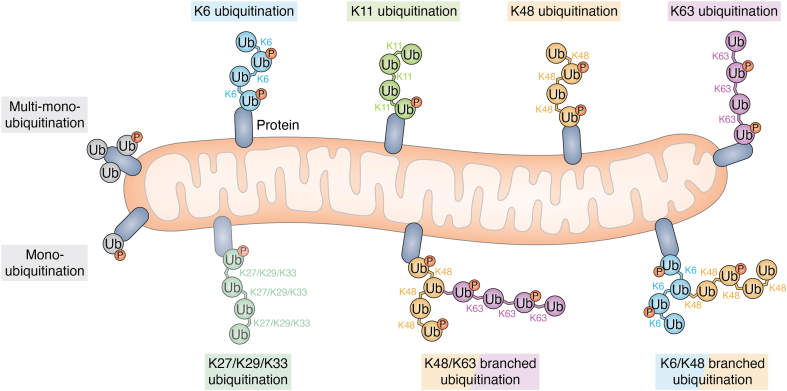
**Diverse pSer65Ub topology on damaged mitochondria.** Damaged mitochondria mainly contain monoubiquitination, multimonoubiquitination, K6, K11, K48, and K63 homotypic linkages, as well as K6/K48 and K48/K63-branched ubiquitin linkages, all of which could contribute to mitophagy. The presence of K27, K29, and K33 has been sporadically detected in proteomics and is considered to be a minor contributor to mitophagy. pSer65Ub, Ser65-phosphorylated ubiquitin.

OMM proteins are subject to Parkin-mediated ubiquitination in response to mitochondrial damage or in disease states (37, 156). In addition to K48 and K63 linkages, Parkin modifies substrates with K11- and K6-linked ubiquitin chains (37, 157). Following mitochondrial depolarization, phosphorylation of ubiquitin at Ser65 (p-Ub) increases by approximately 1100-fold, accompanied by 70-fold and 14-fold increases in K63- and K11-linked ubiquitin chains, respectively (37). Using quantitative middle-down mass spectrometry, Deol *et al.* (2020) demonstrated that Parkin conjugates p-Ub either as a single ubiquitin moiety or at the distal end of poly-ubiquitin chains (157). Notably, when incorporated into poly-ubiquitin chains, p-Ub is preferentially linked through Lys63. Parkin functions as a highly processive and prolific chain-branching E3 ligase, assembling complex poly-ubiquitin architectures containing K6, K11, K48, and K63 linkages. Correspondingly, the mitochondrial DUB USP30 preferentially cleaves branched ubiquitin chains over unbranched chains, underscoring the regulatory significance of chain topology (157). Proteomics studies have also shown that damaged mitochondria are decorated with ubiquitin chains mainly composed of K63, K48, K6, and K11, including mixed and branched architectures (notably K6/K48- and K63/K48-branched chains), with additional lower-abundance of K27/K29/K33 linkages (Fig. 5), reflecting the integration of proteasomal remodeling and autophagy signaling during mitophagy (33, 37, 158, 159, 160, 161, 162, 163, 164). Phosphorylation of these ubiquitin chains by PINK1 adds further complexity to the polyubiquitin architecture (157). Although phospho-poly-ubiquitinated proteins have thus far been detected primarily within mitophagic and autophagic compartments, this spatial restriction does not rule out additional functions for pUb in other aspects of cell physiology.

Together, these findings highlight the extraordinary complexity of the poly-ubiquitin code and demonstrate that PINK1-mediated phosphorylation of ubiquitin further expands the phospho-ubiquitin landscape and its regulatory role in mitochondrial quality control. Antico *et al.* (2021) reported approximately 40-fold and 300-fold increases in phospho-ubiquitin levels in mitochondrial-enriched fractions and whole-cell lysates, respectively (156). Notably, following neuronal damage, only phospho-K63-linked poly-ubiquitin chains were significantly increased. These observations, together with the findings of Deol *et al.* (2020), strongly suggest that mitophagy is primed by the accumulation of phosphorylated K63 poly-ubiquitin chains on mitochondrial proteins. The remarkable tapestry of poly-ubiquitin structures further decorated by phosphorylation implies the existence of specialized receptors that decode the phospho-ubiquitin code.

### pUb biomarker assays are sensitive but not specific enough for clinical application

Multiple ELISA-based detection methods for pUb have been reported and validated using brain samples from *PARK6*/PINK1 KO animals, as well as CSF and serum from humans with normal aging, PD, and AD (130, 139). In 2015, Fiesel *et al.* reported generation of rabbit polyclonal antibodies against pUb (73). The antibody showed a slight preference toward detecting K48-linked pUb chains compared to K63-linked pUb (73). Using this antibody, they showed that pUb-immunoreactive cytoplasmic granules in human brains partially colocalized with mitochondrial and lysosomal markers, as well as total ubiquitin (73). Subsequently the same group reported the evaluation of four different pUb antibodies as capturing agents and five different Ub antibodies as detection agents in developing a highly sensitive sandwich MSD (Meso Scale Discovery) ELISA assay (139). Remarkably, the antibodies performed quite diversely in terms of sensitivity and selectivity. Using one antibody pair (pUb Ab clone E2J6T and total Ub Ab clone P4D1), this ELISA detected femtomolar levels of pUb in plasma samples. Characterization of five additional pUb monoclonal antibodies for various applications including MSD ELISA assay to detect pUb was also reported (165). The performances of these antibodies in MSD ELISA were not significantly different when compared to the previously reported pUb antibody (E2J6T) which was used as a reference. Most of these pUb antibodies preferentially captured K48-linked pUb chains compared to K63-linked pUb chains. In a non-peer-reviewed preprint posted on *Research Square*, Hertz *et al.* (2024) reported detection of pUb in mouse plasma using the SMCxPro platform (Millipore/Sigma) (130). This assay also employed the E2J6T anti-pUb antibody for capture and a fluorescently labeled P4D1 anti-total Ub Ab for detection. Most recently, using the MSD assay, Fiesel *et al.* (2025) evaluated 1500 plasma samples from different cohorts across a spectrum of mild cognitive impairment, AD, or PD (166). Although pUb levels were significantly changed with disease compared to control in certain samples, the measurements in plasma were not sufficiently discriminatory to serve as a robust diagnostic marker. Notably, the same group also evaluated ∼150 CSF samples from two independent case-control series with PD and unexpectedly found that pUb levels were decreased in PD patients compared to controls with better discrimination between control and PD groups (166).

In both MSD and SMCxPro pUb sandwich ELISAs, pUb is captured using a pUb-specific antibody and detected using an antibody against nonphosphorylated, total ubiquitin. Although this approach is sensitive, it results in potential overestimation of pUb levels, as the total Ub antibody could bind to many of the nonphosphorylated ubiquitin moieties present in a polyubiquitin chain that could contain only a single pUb. Such overestimation of pUb levels can impact the utility of pUb as a true biomarker as sometimes the subtle differences between samples may be lost. In addition, the linkage selectivity exhibited by the pUb antibodies used as capturing agents suggests that the pUb structures captured using these antibodies may exclude some of the diverse chain linkage types of pUb, especially the K63 linked pUb chains, resulting in an underestimation of the true pUb status [90]. Another confounding factor is that the current antibody-based capture and detection is far less sensitive for monomeric free pUb compared to polymeric pUb chains. The reason for reduced detection of free pUb has been attributed to the potential steric clashes between two large antibodies (150 kDa each) binding to a small (8 kDa) pUb protein (139). Thus, current methodologies cannot quantitatively estimate pUb due to the lack of tools and the complexity of pUb chain composition and architecture in biological samples. Importantly, human biofluids contain protein phosphatases and DUBs that can significantly influence the accuracy of pUb estimation. Implementation of pUb preservation strategies during biofluid collection, transportation, and storage is likely required to achieve more accurate and reliable pUb quantification. Collectively, these shortcomings in the current pUb estimation technologies impact our ability to accurately evaluate pUb as a biomarker and to study the contributions of pUb to aging and disease pathology. New pUb detection tools that can efficiently capture and detect diverse pUb chain topology will be necessary to circumvent this problem. In addition, pUb tagged individual protein markers might serve as a more specific biomarker for disease than the global levels pUb conjugated to the cellular ensemble of proteins.

## Conclusions and perspectives

There is an important relationship between mitochondrial dysfunction in aging and age-related chronic diseases including neurodegeneration (64, 167, 168). The preservation of mitochondrial functional integrity is critical for sustaining physiological homeostasis during aging key unanswered questions in the PD field include: (1) whether increased pUb levels or impaired mitophagy in PD patients arise from lysosomal dysfunction or from an inability of lysosomal receptors to recognize phospho-polyubiquitinated mitochondrial proteins; and (2) what extent of ubiquitin phosphorylation is associated with the development of sporadic PD. To date, the roles of PINK1 and Parkin in repairing mitochondrial damage, mitobiogenesis, and maintaining mitochondrial dynamics has been widely published and acknowledged (169). Over 300 PINK1 and about 500 parkin human variants have been identified, although not all have established pathogenic effects (135, 170, 171, 172). Identification of phosphorylated ubiquitin as a signature of mitochondrial damage is an important discovery as it allows the development of a blood-based biomarker for identifying diseases characterized by mitophagic dysfunction. However, given the strong correlation of mitochondrial dysfunction in multiple diseases, the level of pUb alone may not be a specific marker. Development of assays that can fully capture the complex and diverse p-poly-Ub protein structures will help establish the utility of pUb as a potential biomarker of dysfunction or disease status. The need for reliable, noninvasive, and specific biomarkers is clear, with the FDA having approved breakthrough device designations for blood tests measuring biomarker levels in AD and PD in the last 5 years, in hopes of facilitating early detection (173, 174). It is becoming increasingly clear, however, that the key to early diagnosis of neurodegenerative disease may lie in multiplexed biomarker tests, measuring disease-specific markers (*e.g.*, α-synuclein, Aβ, pTau) alongside more general markers (*e.g.*, pUb for mitophagic dysfunction). Aided largely by the dedicated efforts of collaborative biomarker-focused programs, such as the Parkinson’s Progression Markers Initiative, a public-private partnership organized by The Michael J. Fox Foundation for Parkinson’s Research, it is our hope that reliable, specific, and tractable biomarker assays for neurodegenerative diseases are on the horizon (175).

## Conflict of interest

K. S., C. R., D. E. S., and T. R. B. are employees of Progenra Inc. M. S. G. has no competing interests.
